# Dementia as a Critical Lens on the Role of Narrative in Medical Training and Practice

**DOI:** 10.1002/hast.4993

**Published:** 2025-09-18

**Authors:** Erin Gentry Lamb, Anita Wohlmann

**Keywords:** dementia, narrative, medical education, metaphor, embodied selfhood, clinical ethics

## Abstract

Narrative holds an important place within medicine and medical education, but an uncritical use of narrative can have troubling consequences for the care of patients who have limited or no capacity for self‐narration, such as those living with dementia. We argue that the guiding principles in the use of narrative within medicine and medical education must be inclusivity and opportunity. We illustrate how medical training can benefit from a more inclusive definition of narrative, and we present a selection of innovative approaches to narrative coming out of literary studies, narrative gerontology, and medical and health humanities that focus on metaphor, embodied selfhood, and critical methods for teaching narrative in medical education. These approaches provide opportunities for medical and health humanities to shape the use of narrative in clinical spaces in critical ways that include and empower more individuals, including medical professionals.

The idea that stories matter for health and medicine is a mainstay of the fields of medical and health humanities, and attention to narrative has increasingly been integrated into medical education. However, the use of narrative within medicine risks further disenfranchising already marginalized populations, such as people living with dementia. “Dementia” is a term that encompasses multiple conditions (with Alzheimer disease being the most prevalent) that affect cognitive abilities such as thought, memory, speech, and behavior. Cultural narratives about dementia position people living with the condition as unreliable narrators of their lives or, in advanced stages, as unable to narrate their lives at all, which contributes to the presumption that dementia leads to a “loss of self.”

In this essay, we use dementia as a lens to examine the use of narrative within medicine and medical education to illustrate how uncritical use of narrative can have troubling consequences for the care of patients who have, depending on the progression of the disease, limited or no capacity for self‐narration in a form that is culturally recognized.^1^ We argue that the guiding principles in the use of narrative within medicine and medical education must be inclusivity and opportunity. We illustrate how medical training can benefit from a more inclusive definition of narrative, and we present a selection of innovative approaches to narrative coming out of literary studies, narrative gerontology, and medical and health humanities that focus on metaphor, embodied selfhood, and critical methods for teaching narrative in medical education. These approaches provide opportunities for medical and health humanities to shape the use of narrative in clinical spaces in critical ways that include and empower more individuals, including medical professionals.^2^

## Narrative in Medicine and Medical Education

Narrative has held an “exceptionally privileged role in medical humanities scholarship” over the twentieth century and into our own.^3^ Narrative has been seen as an essential part of patient and provider experience, crucial to the clinical context, and central to claims that the humanities can improve health care encounters and even outcomes. It has inspired the development of fields like narrative bioethics, narrative medicine, and narrative‐based practice.^4^ Within medical and health humanities and increasingly within medicine and cultures influenced by medicine, the medium of narrative is considered essential both to coping with illness and to practicing effective medicine.

At times, the claims for the value of narrative are so strong that it seems patients and practitioners would be lost without it. For example, in the now classic text *Narrative Medicine: Honoring the Stories of Illness*, Rita Charon suggests, “Without narrative acts, the patient cannot convey to anyone else what he or she is going through. More radically and perhaps equally true, without narrative acts, the patient cannot himself or herself grasp what the events of illness mean. And without telling about or writing about the care of a patient in a complex narrative form, the caregiver might not *see* the patient's illness in its full, textured, emotionally powerful, consequential narrative form.”^5^ At stake here are both the patient's ability to express their “changing sense of self and identity” through illness—a process seen to be “transformative and even therapeutic”—and the provider's ability to treat the patient effectively.^6^

Correspondingly, “narrative competence” is now taught, at least in some medical schools, “as an essential skill in clinical diagnosis and treatment.”^7^ Even more so, across many health care professions, some of the first clinical skills students are taught are how to “elicit the patient's perspective” or “elicit the patient's narrative.”^8^ Students are taught to include direct quotations from the patient into the initial “subjective” portion of their SOAP (subjective, objective, assessment, and plan) notes, which are recorded in the patient's electronic health record. While they may have varying degrees of skill, or patience, students in medicine and other health professions are taught that it is essential to attend to their patients’ narratives to treat them effectively. But what happens when a patient does not have the capacity to form or express their own narrative? Conditions like dementia provide an important challenge to this uncritical emphasis on narrative.

## Dementia and the Negative Consequences of an Emphasis on Narrative

In the advanced stages of dementia, a patient's narrative agency may be limited or barred by the effects of their illness. Narrative agency “refers to [people's] ability to navigate our narrative environments, to use, interpret and reinterpret narratives that are culturally available to us, and to make choices over how we narrate our lives and our relationships.”^9^ Even people with advanced dementia are often able to communicate verbally to some extent, but what they say may not emerge as a fully formed story, may not be clear to the listener regarding time or characters, and may thus be dismissed for incoherency. Exploring the consequences that follow from the perceived lack of narrative agency that accompanies dementia can help one to think more critically about how they conceive of narrative in relation to medical or illness experiences.

Within health care spaces, the problematic understanding of people living with dementia as unreliable narrators may result in communicational neglect and narrative dispossession. As Charlotte Berendonk et al. frame it, if people with dementia “are no longer seen as individuals composing lives and as humans living storied lives … . [T]here are fewer people who deem their experiences as important to listen to.”^10^ This can result in fewer opportunities being made for people living with dementia to share their experiences, or communicational neglect.^11^ It can also lead to family or professional caregivers taking over or controlling those stories. This latter scenario, what Clive Baldwin calls “narrative dispossession,” describes a situation in which “[t]he stories of the people with dementia are hijacked by their caregivers, who assume not just their care, but also the narrative identity of the person with dementia.”^12^ As one practical illustration of why this matters, it is not at all uncommon for a health care professional to address the adult child or the spouse of the person with dementia instead of making space for the person with dementia to speak for themselves and share information their caregiver may not know.

Our stigmatizing cultural narratives of dementia even further constrain people living with the disease because they encourage us to see dementia as the central explanation for everything someone with dementia says or does, overriding other possible interpretations of their words and actions. As Feliciano Villar et al. put it, “Dementia becomes the defining characteristic that gives meaning to their behavior, regardless of their life stories, preferences, needs, expectations, or the actual circumstances that could account for such a behavior from the point of view of the person with dementia.”^13^ When people get an illness like cancer, we expect to see change as they process their experiences. With dementia, we are conditioned to see change only as decline and further loss of the “true self.” Thus, instead of being supported in their own voice, a person diagnosed with dementia may find that their narrative has been overwritten by cultural scripts and they have come to be defined by others’ understanding of the disease. While research on the topic is limited, many medical providers too hold implicit assumptions about and attitudes toward dementia that may affect the way providers interpret the meaning or importance of changing symptoms.

The most important consequence of the perception that someone with dementia is unable to self‐narrate is that others may regard the individual as less than fully human. Such disregard corresponds to the experience among people with dementia of dehumanizing treatment on both structural and individual levels by caregivers, practitioners, and the public. The most extreme forms of such dehumanizing treatment are abuse. Rates of abuse are believed to be significantly under‐reported, but the World Health Organization estimates that one in six people sixty years of age and older experience some form of elder abuse,^14^ and people with dementia are even more likely to experience all forms of elder abuse, with a 2025 systematic review suggesting a global 42.6 percent rate of abuse for people living with dementia in community settings.^15^ Physicians already lack knowledge about and training in detecting elder abuse;^16^ dementia, and a readiness to dismiss the idea that a patient can “speak” for themselves, may further challenge physicians’ capacity for recognizing abuse.

Given these challenges, how can narrative be approached in relation to dementia in a way that benefits the medical experiences of those living with dementia? How can the lens of dementia contribute to more‐critical approaches to narrative within medicine and within medical education?

## Beyond Narrative? Thinking More Inclusively in Medicine and Medical Education

One challenge in thinking more critically about narrative is that there are debates, particularly within the fields of medical and health humanities, about how broadly or narrowly “narrative” should be defined. On one side of the debate, narratologists typically advocate for more‐precise differentiations between *narrative* and *story* and *plot*, suggesting that more clearly demarcated definitions of “narrative” prevent the term from becoming meaningless if narrative is used for all kinds of expressions. In contrast, others such as those in narrative medicine tend to operate with a more inclusive understanding of “narrative” that encompasses a whole range of other forms of expression, such as performance, dance, and visual art.^17^ We tend to agree with Stella Bolaki that “there is room to challenge *and* expand narrative's conception and role within the medical humanities field.”^18^ Thus, instead of entering into this conversation around demarcations, what matters most is attending to the priorities of inclusivity and openness in the *use* of narrative. The lens of dementia pushes one to explore alternative forms of expression and critical tools for interpreting these forms.

In this spirit, Angela Woods speaks to the value of “non‐narrative forms of communications” such as metaphor, phenomenology, and photography.^19^ Additionally, several scholars have called for attending to expressions of meaning that do not share traditional features of narratives such as the “lyric mode,”^20^ the episodic,^21^ or seriality.^22^ Narratologists like H. Porter Abbott, Gerald Prince, and Robyn R. Warhol have explored phenomena like the “disnarrated,” the “unnarratable,” and “non‐narration.”^23^ There is also room to rethink narrative outside of Eurocentric traditions in ways that foster inclusion.^24^ Speaking in particular of Indigenous narrative forms, which often forgo the canonical hero narrative and instead “meander” and “draw us in to another reality,” Nicola Grove argues that the valuing of “open‐endedness and provisionality” can make more recognizable “the abilities and contribution of those members of our community who are marginalized by a focus on autonomous, structurally complete discourse, neatly packaged in a coherent sequence.”^25^ Scholars like Claire McKechnie argue that it is impossible to move “beyond” narrative, asserting that “in order to be interpreted (indeed interpretable),” forms “of non‐narrative representation and communication … in fact require a narrative response.”^26^ We can envision many non‐narrative ways of responding to communication: sitting in silence, praying, hugging, and so forth. Yet we acknowledge that meaning making—internal processing of another's words and behaviors—is generally considered a narrative process, and it requires active effort on the part of those who seek to understand another's narrative.

To explore critical approaches to the use of narrative in medicine and medical education that may encourage better experiences for people living with dementia, we turn first to metaphor. Catherine Belling claims that metaphor has the capacity to yank people “outside the current of plot” through tiny shocks of surprise and flashes of immediate, seemingly inevitable insight.^27^ Such jolts to consciousness are particularly prevalent in new, vivid metaphors, which are often associated with atemporality and sensory experiences rather than with causality and linearity.^28^ For example, the metaphor “a head full of honey”—as used in the German feature film *Honig im Kopf* (2014)^29^—compares the experience of dementia with the visceral experience of the stickiness and thickness of honey. In other words, the brain that used to think on its feet now feels impenetrable and heavy; the person who once swiftly navigated through the world is now slowed down, impeded and stuck. Being sweet and nourishing, honey also has many pleasant associations, and all this taken together produces a metaphor that feels fresh and unexpected, enabling a host of ambiguous connotations that may but need not be linked to a narrative to suggest meaning.^30^

Still, metaphor need not be seen as a foil to narrative. Some scholars even describe metaphors as micronarratives or mininarrations,^31^ and some metaphors such as “life is a journey” or “life is a story” are hard to disentangle from the narrative potential they carry. Metaphors for dementia such as a “ship lost at sea”^32^ and “exile” and “emigration”^33^ provide many more opportunities to help patients explore the narrative challenges that a dementia diagnosis may create for their identity than do prevailing popular zombie metaphors depicting people living with dementia as “the living dead.”^34^ There is value in analyzing metaphors for dementia—such as a “head full of honey,” a “ship at sea,” and “exile”—*as* metaphors and thus as expressions with distinct rules and affordances. According to Paul Ricœur, a focus on the non‐narrative side of a metaphor allows one to consider the tension between what is and what is not that every metaphor calls forth,^35^ enabling a “stereoscopic vision” through which depth perception becomes possible.^36^ In other words, metaphors invite one into an imaginative, experimental space where subjective experiences and personal meanings are simultaneously true and untrue and where logical reasoning and narrative coherence are temporarily suspended. A medical provider skilled in metaphor analysis may be able to help a patient with dementia recognize the multiple connotations—both positive and negative—that various metaphors may carry. Training in medical education that pays attention to the affordances and limitations of metaphors both within and outside of narrative acknowledges a broader range of meaningful expressions.

Another way to better account for the experiences of individuals with dementia is through attention to the body. Pia Kontos has developed a theory of *embodied selfhood* that enables thinking about identity and personhood in ways that do not rely on the ability to self‐narrate. She makes the case that “selfhood is embodied and characterised by an observable coherence and capacity for improvisation that is sustained at a pre‐reflective level by the primordial as well as the socio‐cultural significance of the body.”^37^ Through her ethnographic observations, Kontos explores the nondiscursive ways that selfhood is produced through appearance, social etiquette, caring, dancing, and gestural communication, insisting that “the body is a fundamental source of selfhood that does not derive its agency from a cognitive form of knowledge.”^38^ Kontos argues that considering embodied selfhood within dementia care could lead to the development of “a new ethic of care” that encourages caregivers to more closely sensitize themselves to ways that the body can express the self and, in so doing, to foster interpersonal relationships between caregivers and care recipients.^39^ While the care providers here may be using their own narrative frame to make meaning of these behaviors, they are encouraged to ground that narrative in a closer reading of the body and actions of someone with dementia, rather than hijack the person's narrative. Although medical professionals in their relatively brief clinical encounters with people living with dementia may have less opportunity to observe and interpret such behaviors, they have plenty of opportunity to raise thoughtful questions of the caregivers about how they understand the experiences of the person living with dementia. What gestures, expressions, or actions indicate that the person is happy, anxious, hungry? What specific behaviors have existed since well before diagnosis, what new behaviors have appeared, and how does the caregiver interpret these and why? Such questions could help train caregivers to better attend to embodied communication and encourage them to ground their narratives in details *from* the person living with dementia.

Attention both to metaphor and to embodied communication could be part of critical approaches to teaching about narrative in medical education. Currently, there is no guarantee that narrative is a part of the curriculum beyond an uncritical emphasis on “the patient's narrative.” When narrative does enter the curriculum, at least within the United States, it is most often approached through some version of narrative medicine that focuses, broadly, on teaching students how to elicit, interpret, and act upon patients’ stories. Narrative medicine approaches may take different forms, but often students are asked to apply skills of literary analysis (close attention to perspective, form, voice, mood, and motion) to short literary texts—usually, with the explicit idea that such methods of analysis can be transferred into better “reading” patients, or narrative competence—and frequently to write in response to a specific prompt, sharing these efforts with others in a small‐group setting. Attention to narrative is often relegated to educators positioned in medical or health humanities or social medicine (if medical schools include these disciplines), though the effectiveness of such activities may depend on the skill of small‐group facilitators, who may or may not have training with narrative and literary analysis.

We offer two examples, drawing from well‐known narrative medicine texts, to illustrate how current approaches to narrative medicine in medical education could be used to raise critical questions about how narrative may work differently for people living with dementia.

James Phelan's *Narrative Medicine: A Rhetorical Rx* (2023) defines narrative as “somebody telling somebody else on some occasion and for some purpose(s) that something happened.”^40^ This definition does not emphasize coherence or linearity, and Phelan's criteria for attentive analysis focus on the speaker (or author), audiences, character, time, space, perspective, and voice. With this framework, Phelan opens the study of narratives to a wide range of expressive forms, which he exemplifies through a “seven‐word story”—or example of flash fiction—by Joyce Carol Oates: “‘Widow's First Year’: I kept myself alive.”^41^

By focusing on the teller, audience, and purpose of this “story” rather than the logic of events, temporal progression, and coherence, Phelan teases out the semantic and affective depths of this “story about grief.”^42^ Guiding medical students through an interpretation of this story demands a high commitment and careful imagination on the part of the interpreter, who has to fill in gaps and construct meaning from very little information while remaining modest about their assumptions and cautious about the risks of appropriation that such an endeavor may bring.^43^ Drawing attention to the potentials and pitfalls of every interpretative act allows educators to link Oates's story to the ways in which health care professionals are sometimes required to make assumptions and fill gaps in the stories of patients with dementia who may have limited communication skills. The engagement with the literary text thus provides an opportunity for medical students to question the assumptions they hold about dementia, the possible strategies they might use for interpreting experiences based on little information, and the risks that may come from relying only on a caregiver's version of the patient's experience.

Another key narrative medicine text that might be critically incorporated into medical education is *The Principles and Practice of Narrative Medicine* (2017), by Charon and the Narrative Medicine group at Columbia University. Even though the text mentions dementia only in passing, it offers multiple opportunities for including dementia in its approach. Craig Irvine and Danielle Spencer illustrate this point in their close reading^44^ of David Ferry's poem “Soul,” which opens with “What am I doing inside this old man's body?”^45^ The poem describes, in disconnected associations, a sense of alienation and estrangement that could easily be related to dementia or aphasia (which Irvine and Spencer briefly acknowledge in their discussion).^46^ While poets and writers can be extraordinarily eloquent (which people living with dementia may not or no longer be), written works that feature disconnection or stream‐of‐consciousness can be useful in the training of health care professionals, reminding them that “broken” stories may not always require fixing^47^ and that there are other meaningful ways of using language and expressing the complicated nature of human consciousness and cognition.

## Toward a Critical Attention to Narrative in Medicine and Medical Education

Narrative is certainly “consequential” for medicine and medical education, but too uncritical a use of narrative disenfranchises those living with dementia or others with serious cognitive or communication impairments. Thinking through the challenges that dementia presents to the use of narrative in medicine enables students and professionals in medicine and related fields to think more critically and broadly about how their understanding of narrative affects whom they see as capable of narrating and how centering those who seem to have the least narrative agency can help in developing better narrative practice. The foundation of better narrative practice for physicians must be laid in medical education. Attending to metaphor, embodied selfhood, and critical approaches to narrative in medical education is key to recognizing the importance of many forms of expression in illness contexts and to making space for those who are unable to self‐narrate in conventional terms.

In addition to skill, intention is a necessity. While interpreting someone else's story inevitably raises ethical issues of authority and authenticity (among others)—requiring a self‐reflexivity and humility that should be part of any approaches to narrative in medicine and medical education^48^—the interpretive interactions involved are not so far distant from the “interdependent and intersubjective nature of our lives and identities” writ large.^49^ As Rebecca Bitenc argues, “People with dementia continue to make coherent, that is[,] ‘intelligible’ identity claims. Whether their claims are recognised or not depends, however, less on the formal criteria of their identity narratives than on the willingness and ability of the people around them to engage with these claims.”^50^ Bitenc's words serve as a reminder that efforts to think differently and more critically about people's experiences living with dementia and about cultural narratives of dementia—in ways that insist on centering the selfhood and humanity of people living with dementia—must also be part of medical education and medical practice.

We have argued for the importance of more critical attention to narrative in medicine and medical education that includes skills and intention, and that is guided by the principles of inclusivity and opportunity. The hoped‐for stakes of such an approach are that patients living with dementia can have their expressive agency recognized and supported, leading both to improved individual lived experiences and to shifts in larger cultural narratives of dementia.

## References

[1] Western narrative is a form that seems to demand potentially problematic criteria: “sequence, succession, causality … closure.” P. Tammi, “Against Narrative (‘A Boring Story’),” Partial Answers: Journal of Literature and the History of Ideas 4, no. 2 (2006): 19–40, at 22. Dominant approaches to narrative in medicine privilege recovery and the completed journey—what Arthur Frank in his typology of illness narratives refers to as the “restitution” or “quest narratives.” A. W. Frank, *The Wounded Storyteller: Body, Illness, and Ethics,* 2nd ed. (Chicago, IL: University of Chicago Press, 2013). Dementia—a typically age-associated, progressive, ultimately terminal condition without effective treatments—makes it impossible to follow the arc of recovery and a return to the normal of the restitution narrative. The quest narrative—a frame that helps ill people reorder their experience, attach new meaning to it, and experience themselves as agents of their own life story—is likewise problematic, as it relies upon a decidedly Western conceptualization of the self as an autonomous agent that is challenged by the decreasing cognitive capacity of advanced stages of dementia. A. Woods, “The Limits of Narrative: Provocations for the Medical Humanities,” *Medical Humanities* 37, no. 2 (2011): 73-78, at 74. Medicine's notion of “the illness narrative” leaves little room to see dementia as anything other than what Frank dubs a “chaos narrative”—fragmented and disjointed; this provides individuals receiving a diagnosis of dementia with little, if any, hope for meaningful existence ahead.

[2] Also see R. Ahlzén and his plea for more-modest approaches to the potentials of narratives in the context of health care in “Narrativity and Medicine: Some Critical Reflections,” Philosophy, Ethics, and Humanities in Medicine 14, no. 9 (2019): 1–10. 10.1186/s13010-019-0078-3 PMC6633324 31307498

[3] Woods , “The Limits of Narrative,” 73.

[4] M. Montello ed., “Narrative Ethics: The Role of Stories in Bioethics,” special report, Hastings Center Report 44, no. 1 (2014); R. Charon et al., eds., *The Principles and Practice of Narrative Medicine* (London: Oxford University Press, 2017); J. Launer, *Narrative-Based Practice in Health and Social Care: Conversations Inviting Change*, 2nd ed. (London: Routledge, 2018).

[5] R. Charon , Narrative Medicine: Honoring the Stories of Illness (Oxford: Oxford University Press, 2006), 13.

[6] Woods , “The Limits of Narrative,” 73.

[7] Ibid., 73.

[8] For example , “[e]licit the [p]atient's [p]erspective” is the second habit of the “The Four Habits Model” proposed in R. M. Frankel and T. Stein, “Getting the Most out of the Clinical Encounter: The Four Habits Model,” Journal of Medical Practice Management 16, no. 4 (2001): 184–91. More recently, “[e]licit the patient narrative” is a key part of phase II of the REDE Model of Healthcare Communication developed by the Cleveland Clinic in 2014. A. K. Windover et al., “The REDE Model of Healthcare Communication: Optimizing Relationship as a Therapeutic Agent,” *Journal of Patient Experience* 1, no. 1 (2014): 8-13. 28725795 10.1177/237437431400100103 PMC5513605

[9] H. Meretoja , “Narrative Agency, Life Writing, and the Experience of Illness,” talk at the Oxford Centre for Life Writing, May 5, 2020, https://oclw.web.ox.ac.uk/article/narrative-agency-life-writing-and-the-experience-of-illness.

[10] C. Berendonk et al., “A Narrative Care Approach for Persons Living with Dementia in Institutional Care Settings,” International Journal of Older People Nursing 15, no. 1 (2020): doi:10.1111/opn.12278. 31577388

[11] F. Villar , R. Serrat , and S. Bravo-Segal , “Giving Them a Voice: Challenges to Narrative Agency in People with Dementia,” Geriatrics 4, no. 20 (2019): 1–9, at 4-5. 10.3390/geriatrics4010020 PMC6473304 31023988

[12] Ibid., 5.

[13] Ibid., 5.

[14] World Health Organization , “Abuse of Older People,” June 15, 2024, https://www.who.int/news-room/fact-sheets/detail/abuse-of-older-people

[15] Y. Shen et al., “Prevalence of Elder Abuse and Neglect of Persons with Dementia in Community Settings: A Systematic Review and Meta-analysis,” Gerontology 71, no. 5 (2025): 400–416. 10.1159/000543804. 40552876

[16] B. Ratnakaran and S. S. Anil , “A Systematic Review of Knowledge of Physicians about Abuse of Older Adults,” supplement, American Journal of Geriatric Psychiatry 28, no. 4 (2020): S104–5.

[17] D. Spencer , “Narrative Medicine,” in Routledge Companion to Philosophy of Medicine, ed. M. Solomon , J. R. Simon , and H. Kincaid (New York: Routledge, 2017), 372–82, at 374.

[18] S. Bolaki , Illness as Many Narratives: Arts, Medicine and Culture (Edinburgh: Edinburgh University Press, 2016), 6.

[19] Woods , “The Limits of Narrative,” 73.

[20] C. Belling , “A Happy Doctor's Escape from Narrative: Reflection in *Saturday* ,” Medical Humanities 38, no. 1 (2012): 2–6. 22345587 10.1136/medhum-2011-010129

[21] S. Wasson , “Before Narrative: Episodic Reading and Representations of Chronic Pain,” Medical Humanities 44, no. 2 (2018): 106–12; G. Strawson, “Against Narrativity,” *Ratio* 17, no. 4 (2004): 428-52. 29305389 10.1136/medhum-2017-011223 PMC6031271

[22] A. Wohlmann and M. Harrison , “To Be Continued: Serial Narration, Chronic Disease, and Disability,” Literature and Medicine 37, no. 1 (2019): 67–95. 31402343 10.1353/lm.2019.0002

[23] H. P. Abbott , “Unnarratable Knowledge: The Difficulty of Understanding Evolution by Natural Selection,” in *Narrative Theory and the Cognitive Sciences,* ed. D. Herman (Chicago: University of Chicago Press, 2003), 143-62; G. Prince , “The Disnarrated,” Style 22, no. 1 (1988): 1–8; R. R. Warhol, “Neonarrative; or, How to Render the Unnarratable in Realist Fiction and Contemporary Film,” in *A Companion to Narrative Theory,* ed. J. Phelan and P. J. Rabinowitz (Malden, MA: Blackwell, 2008), 220-31.

[24] Additionally, we should acknowledge that Western literary theory—through modernism to postmodernism to world literature—already accepts that what counts as narrative far exceeds criteria such as sequence, causality, and closure.

[25] N. Grove , “Story, Agency, and Meaning Making: Narrative Models and the Social Inclusion of People with Severe and Profound Intellectual Disabilities,” Journal of Religion, Disability & Health 16, no. 4 (2012): 334–51, at 343, 345.

[26] C. C. McKechnie , “Anxieties of Communication: The Limits of Narrative in the Medical Humanities,” Medical Humanities 40, no. 2 (2014): 119–24, at 120. 24869471 10.1136/medhum-2013-010466

[27] Belling , “A Happy Doctor's Escape from Narrative,” 5. See also J. Wood , How Fiction Works (New York: Farrar, Straus & Giroux, 2008), 202, 209.

[28] A. Wohlmann , Metaphor in Illness Writing: Fight and Battle Reused (Edinburgh: Edinburgh University Press, 2022), 105ff.

[29] For a critical discussion of the movie, see S. Horlacher and F. Röber , “Of Bees, Boobies and Frank Sinatra: Masculinity and Alzheimer's in Contemporary European Film Comedies,” in Ageing Masculinities, Alzheimer's and Dementia Narratives, ed. H. Hartung, R. Kunow, and M. Sweney (London: Bloomsbury Academic, 2022), 105-24.

[30] For another example of a non-narrative approach to dementia that reads the expressions of a person with dementia as metaphors, see A. Swinnen , “Dementia in Documentary Film: *Mum* by Adelheid Roosen,” Gerontologist 53, no. 2 (2013): 113–22, at 120. 22539659 10.1093/geront/gns061

[31] P. Eubanks , “The Story of Conceptual Metaphor: What Motivates Metaphoric Mappings?,” Poetics Today 20, no. 3 (1999): 419–42.

[32] G. M. Carney et al., “‘Sometimers, Alzheimer's? I love that! That's definitely me’: Readers’ Responses to Fictional Dementia Narratives,” Gerontologist 63, no. 10 (2023): 1610–18, at 1615. 37170858 10.1093/geront/gnad055 PMC10724039

[33] M. Schrage-Früh , “Stories of Exile and Home: Dementia and Masculinity in Arno Geiger's Der alte König in seinem Exil and I. Maleney's *Minor Monuments,*” in *Ageing Masculinities, Alzheimer's and Dementia Narratives,* ed. H. Hartung, R. Kunow, and M. Sweney (London: Bloomsbury Academic, 2022), 145-61.

[34] S. M. Behuniak , “The Living Dead? The Construction of People with Alzheimer's Disease as Zombies,” Ageing & Society 31, no. 1 (2011): 70–92.

[35] P. Ricœur , The Rule of Metaphor: The Creation of Meaning in Language, 3rd ed. (London: Taylor and Francis, 2004), 293.

[36] P. Ricœur , Interpretation Theory: Discourse and the Surplus of Meaning (Fort Worth, TX: Texas Christian University Press, 1976), 50, 56.

[37] P. Kontos , “Ethnographic Reflections on Selfhood, Embodiment and Alzheimer's Disease,” Ageing & Society 24, no. 6 (2004): 829–49, at 831.

[38] P. Kontos , “Embodied Selfhood in Alzheimer's Disease: Rethinking Person-Centred Care,” Dementia 4, no. 4 (2005): 553–70, at 553.

[39] Ibid., 565.

[40] J. Phelan , Narrative Medicine: A Rhetorical Rx (New York: Routledge, 2023), 4.

[41] Ibid., 8.

[42] Ibid., 5.

[43] S. DasGupta , “Narrative Humility,” Lancet 371 (2008): 980–81; C. Irvine, “The Other Side of Silence: Levinas, Medicine, and Literature,” *Literature and Medicine* 24, no. 1 (2005): 8-18. 16134833 10.1353/lm.2005.0027

[44] Charon proposes close reading as a “signature method” that focuses the reader's attention on the workings of time, space, voice, and metaphor (or, as in Charon's 2006 monograph, the analysis of frame, form, time, plot, and desire).

[45] Charon et al., The Principles and Practice of Narrative Medicine, 105.

[46] Ibid., 105–6.

[47] H. Brody , “‘My Story Is Broken; Can You Help Me Fix It?’ Medical Ethics and the Joint Construction of Narrative,” Literature and Medicine 13, no. 1 (1994): 79–92. 8007732 10.1353/lm.2011.0169

[48] DasGupta , “Narrative Humility.”

[49] R. A. Bitenc , “‘No Narrative, No Self’? Reconsidering Dementia Counter-Narratives in *Tell Mrs. Mill Her Husband Is Still Dead* ,” Subjectivity 11, no. 2 (2018): 128–43, at 142.

[50] Ibid., 141.

